# Dr. Frank E. Dewey

**Published:** 1910-01

**Authors:** 


Dr. Frank E. Dewey, of Peterboro, N. Y., died at Oneida, suddenly December 6, 1909, of heart disease, aged 60 years. He graduated at the University of Buffalo, Medical Department in 1873.

